# LINC00942 inhibits ferroptosis and induces the immunosuppression of regulatory T cells by recruiting IGF2BP3/SLC7A11 in hepatocellular carcinoma

**DOI:** 10.1007/s10142-024-01292-4

**Published:** 2024-02-14

**Authors:** Dong Jin, Yongfeng Hui, Di Liu, Nan Li, Junzhi Leng, Genwang Wang, Qi Wang, Zhenhui Lu

**Affiliations:** 1 Department of Hepatobiliary Surgery, Ningxia Medical University General Hospital, 804 Shengli South Street, Xingqing District, Yinchuan, 750004 Ningxia China; 2 Department of Hepatobiliary Surgery, Shekou Shenzhen People’s Hospital, 36 Shekou Industrial 7 Road, Nanshan District, Shenzhen, 518067 Guangdong China

**Keywords:** Hepatocellular carcinoma (HCC), LINC00942, SLC7A11, IGF2BP3, Ferroptosis

## Abstract

Hepatocellular carcinoma (HCC) is a common malignant tumor with a high recurrence rate and a poor prognosis. Long intergenic nonprotein coding RNA 942 (LINC00942) is reported to be related to ferroptosis and the immune response in HCC and serves as an oncogene in various cancers. This research aimed to explore the contribution of LINC00942 in HCC progression. Functional assays were used to evaluate the functional role of LINC00942 in vitro and in vivo. Mechanistic assays were conducted to assess the association of LINC00942 with insulin-like growth factor 2 mRNA binding protein 3 (IGF2BP3) and solute carrier family 7 member 11 (SLC7A11) and the regulatory pattern of LINC00942 in HCC cells. LINC00942 was found to exhibit upregulation in HCC tissue and cells. LINC00942 facilitated HCC cell proliferation, suppressed ferroptosis, and converted naive CD4^+^ T cells to inducible Treg (iTreg) cells by regulating SLC7A11. Furthermore, SLC7A11 expression was positively modulated by LINC00942 in HCC cells. IGF2BP3 was a shared RNA-binding protein (RBP) for LINC00942 and SLC7A11. The binding between the SLC7A11 3′ untranslated region and IGF2BP3 was verified, and LINC00942 was found to recruit IGF2BP3 to promote SLC7A11 mRNA stability in an m6A-dependent manner. Moreover, mouse tumor growth and proliferation were inhibited, and the number of FOXP3^+^CD25^+^ T cells was increased, while ferroptosis was enhanced after LINC00942 knockdown in vivo. LINC00942 suppresses ferroptosis and induces Treg immunosuppression in HCC by recruiting IGF2BP3 to enhance SLC7A11 mRNA stability, which may provide novel therapeutic targets for HCC.

**Supplementary information:**

The online version contains supplementary material available at 10.1007/s10142-024-01292-4.

## Introduction

Primary liver cancer ranked as the sixth among frequently diagnosed tumors and the third among causes of cancer-related mortality globally in 2020 (Sung et al. 2021). Hepatocellular carcinoma (HCC) is the most common primary liver cancer and appears frequently in cirrhosis patients (Forner et al. 2018). Surgical resection, transplantation, ablation, transarterial chemoembolization, and the tyrosine-kinase inhibitors sorafenib, Lenvatinib, and regorafenib can improve the survival of patients. During the past several decades, although the 5-year survival rate has increased in advanced HCC patients, the recurrence rate is approximately 80% (Lin et al. 2020a). Thus, it is necessary to find novel biomarkers for therapy of HCC.

Ferroptosis is an iron-dependent form of regulated cell death that occurs via the lethal accumulation of intracellular lipid reactive oxygen species (ROS) (Dixon et al. 2012). In contrast to autophagy, necroptosis, and other forms of regulated cell death, ferroptosis induces cell death independent of DNA damage (Lei et al. 2020). Moreover, ferroptosis dysregulation gets involvement in the progression of various cancers as a risk factor that promotes tumor growth. For instance, ITGB2-AS1 promotes non-small-cell lung cancer cisplatin resistance via repressing p53-mediated ferroptosis (Chen et al. 2023). Linc00976 exerts a vital role in elevating cholangiocarcinoma tumorigenesis and metastasis and inhibiting ferroptosis (Lei et al. 2022). The impact of ferroptosis underlying HCC has also been demonstrated (Manz et al. 2016; Mou et al. 2019; Nie et al. 2018). Activated CD8^+^ T cells enhance tumor cell ferroptosis, which leads to advancements in immunotherapy (Wang et al. 2019b). Enhanced ferroptosis induces immunosuppression in glioblastoma via the activation and infiltration of immune cells (Liu et al. 2022). Ferroptosis is also implicated in the construction of the immunosuppressive HCC tumor microenvironment (Hu et al. 2022). Therefore, inducing ferroptosis may be a potentially effective option for HCC treatment.

Long noncoding RNAs are transcripts longer than 200 nucleotides with no protein coding abilities. LncRNAs are implicated in various physiological and pathological processes via diverse mechanisms (Schmitz et al. 2016). Many lncRNAs have been demonstrated to regulate HCC progression (Wang et al. 2017b, 2019a; Wu et al. 2021). Long intergenic noncoding RNA 00942 (LINC00942) is a cancer promoter in various tumors. For example, LINC00942 facilitates growth of lung adenocarcinoma by targeting miR-5006-5p to upregulate FZD1 (Wang et al. 2021). LINC00942 enhances gastric cancer chemoresistance by inhibiting apoptosis and inducing stemness (Zhu et al. 2022). Moreover, LINC00942 is also reported to be correlated with ferroptosis and the immune response in HCC, and its upregulation predicts adverse prognosis in HCC patients (Xu et al. 2021b). Intriguingly, LINC00942 has also been validated as an immune-related gene in HCC (Xu et al. 2021a). It has been demonstrated that lncRNAs can exert biological functions by sequestering miRNAs or recruiting RNA binding proteins (RBPs) to regulate miRNAs (Gong et al. 2023; Wang et al. 2023b). However, explicit underlying mechanism by which LINC00942 regulates ferroptosis and the immune response in HCC remains indistinct.

This research attempted to investigate the contribution of LINC00942 in ferroptosis and immune response underlying HCC. We hypothesized that LINC00942 might promote HCC progression by suppressing ferroptosis and inducing the immunosuppression of HCC Tregs. This research may provide novel biomarkers for HCC therapy.

## Methods

### Reagents

Dulbecco’s modified Eagle’s medium (DMEM) containing fetal bovine serum (FBS), penicillin‒streptomycin, and actinomycin D was purchased from Sigma-Aldrich (USA). TRIzol reagent, Lipofectamine 3000, anti-insulin-like growth factor 2 mRNA binding protein 3 (IGF2BP3) primary antibody, bicinchoninic acid (BCA) kit, electrochemiluminescence (ECL) kit, BODIPY-C11, and SYBR Premix Ex Taq were purchased from Thermo Fisher Scientific (USA). The CCK-8 reagent was purchased from Dojindo Laboratories (Kumamoto, Japan). EdU reagent and RIPA lysis buffer were purchased from Beyotime (Shanghai, China). Anti-solute carrier family 7 member 11 (SLC7A11), anti-m6A, anti-U2AF2, anti-IgG antibodies, and an iron assay kit were purchased from Abcam (Shanghai, China). A fluorescence in situ hybridization (FISH) kit was purchased from RiboBio (Guangzhou, China). The Magna MeRIP m6A kit was purchased from Millipore (USA). The Magnetic RNA Protein Pull-Down Kit was purchased from Pierce (USA).

### Cell culture and treatment

HCC cell lines (HepG2, HuH-7, Li-7) and the normal adult liver epithelial cell line THLE-2 were obtained from American Type Culture Collection (ATCC, Manassas, VA, USA) and incubated in DMEM containing 10% FBS and 1:100 penicillin‒streptomycin in a humidified incubator at 37 °C with 5% CO_2_. The mRNA stability was assessed with 50-mM actinomycin D treatment for another 6, 12, 18, and 24 h (Shen et al. 2023). Finally, RNA was isolated from HCC cells using TRIzol reagent and quantified using qRT-PCR with glyceraldehyde-3-phosphate dehydrogenase (GAPDH) as the control.

### Cell transfection

Short-harpin RNAs (shRNAs) against LINC00942 (sh-LINC00942-1, sh-LINC00942-2) and IGF2BP3 (sh-IGF2BP3-1, sh-IGF2BP3-2), pcDNA3.1/LINC00942, and pcDNA3.1/SLC7A11 were provided by Genomeditech (Shanghai, China). HCC cells were grown in 6-well plates and transfected with the above plasmids with Lipofectamine 3000. After transfection for 48 h, the HCC cells were collected for further study.

### Cell counting kit-8 assay

A CCK-8 assay was conducted to assess HCC cell viability s (Zhao et al. 2023). The transfected cells (1.5 × 10^3^ cells/well) were grown in 96-well plates for 48 h. Next, CCK-8 reagent (10 µL) was added for incubation for 2 h. The viability was determined by detecting the absorbance at 450 nm using a microplate reader (Bio-Rad, Hercules, CA, USA). There are three biological replicates for CCK-8 assay.

### EdU staining

An EdU kit was used to detect HCC cell proliferation (Hou et al. 2023). The transfected HCC cells were grown in 24-well plates at 5 × 10^4^ cells/well. EdU reagent was added according to the manufacturer’s instructions. After washing with PBS, the cells were stained with DAPI for 5 min. Finally, EdU-positive cells were observed under a fluorescence microscope and quantified using ImageJ software in five randomly chosen visual fields. There are three biological replicates for EdU staining assay.

### qRT-PCR

Total RNAs were isolated from HCC cells using a TRIzol reagent kit and reverse transcribed into complementary DNAs (cDNAs). qRT-PCR was performed with SYBR Premix Ex Taq in the StepOne Plus system. The 2^−ΔΔCq^ method was used for RNA expression quantification with GAPDH as the reference gene, whose stability was determined by a computational software RefFinder (Xie et al. 2012). The sequences of the primers were as follows: LINC00942: F: 5′-GTTTCCCTGGAAACACCAC-3′, R: 5′-TTGAACATGAAGGCAGGTG-3′; SLC7A11: F: 5′-CATCGTCCTTTCAAGGTGC-3′, R: 5′-ATAGAGGGAAAGGGCAACC-3′; IGF2BP3: F: 5′-GAACACTGACTCGGAAACTG-3′, R: 5′-TTCAGTTTGTCTAGTGCTTGTC-3′; ABCB6: F: 5′-TTCGTCGTGCTATGAACAC-3′, R: 5′-TGTAATACTTCACCGTCTCGA-3′; and GAPDH: F: 5′-CCTCCTGTTCGACAGTCAG-3′, R: 5′-CATACGACTGCAAAGACCC-3′. There are three biological replicates and three technical replicates for *qRT-PCR.* There are three biological replicates for qRT-PCR.

### Western blotting

Total protein in HCC cells was isolated with radioimmunoprecipitation assay (RIPA) buffer, and concentration of proteins received determination by a BCA kit. After loading 50 μg of protein for each sample on a polyacrylamide gel for electrophoresis, the proteins were electrotransferred onto nitrocellulose membranes. Next, the membranes received blocking with 5% BSA and then incubated with primary antibodies, including anti-SLC7A11 (ab175186, 1:1000) and anti-IGF2BP3 (PA5-86040, 1:1000), at 4 °C overnight with β-actin as the internal control. Then, membranes received incubation with secondary antibody for another 2 h (Hou et al. 2023). The protein band signal was imaged with an ECL kit and quantified by ImageJ software. There are three biological replicates for western blotting.

### RNA immunoprecipitation

The interaction of LINC00942 and the SLC7A11 3′UTR with corresponding RBPs was explored using a RNA immunoprecipitation (RIP) assay with the Magna RIP RNA-Binding Protein Immunoprecipitation Kit (Hou et al. 2023). Briefly, HCC cells were lysed with RIPA buffer for 5 min. Moreover, cells received incubation with antibodies against U2AF2, IGF2BP3, and m6A and negative control IgG conjugated with magnetic beads for immunoprecipitation at 4 °C overnight. Finally, proteinase K was used to treat the samples, and RNAs was isolated and subjection to qRT-PCR. The SLC7A11 3′UTR is shown in the supplementary file. There are three biological replicates for RIP assay.

### RNA pulldown

An RNA pulldown assay received performance to further analyze the binding between LINC00942 and IGF2BP3 in HCC cells. The Magnetic RNA Protein Pull-Down Kit was used for RNA pulldown assay according to the manufacturer’s guidance. HCC cell lysates were cultured with biotinylated LINC00942 (RiboBio) and streptavidin-agarose beads. Finally, the proteins were washed and eluted from the beads, separated by SDS-PAGE, and further analyzed using western blotting. There are three biological replicates for RNA pulldown assay.

### RNA fluorescence in situ hybridization

A FISH kit was used to detect distribution of LINC00942 and IGF2BP3 in HCC cells (Huang et al. 2023). The probes for LINC00942 and IGF2BP3 were provided by RiboBio. After treating the HCC cells with 4% paraformaldehyde and 0.5% Triton X-100 for fixation and permeabilization for 30 min at 4 °C, the HCC cells that underwent prehybridization received culture with LINC00942 and IGF2BP3 probes overnight at 37 °C. DAPI was used to counterstain cell nuclei. A fluorescence microscope was used to take photos and analyze localization of LINC00942 and IGF2BP3. There are three biological replicates for FISH assay.

### MeRIP-qPCR

m6A RNA immunoprecipitation (MeRIP) was performed as previously described using a Magna MeRIP m6A kit (Yang et al. 2020). There are three biological replicates for MeRIP.

### Flow cytometry

Flow cytometry analysis was performed for lipid ROS measurement (Yu et al. 2023). HCC cells were grown in 6-well plates at 2 × 10^5^ cells/well. After treatment with 2 µM erastin or an equal volume of DMSO for 12 h, the culture medium received clearing and washing with PBS. Next, HCC cells received staining with BODIPY-C11 (5 µM) for 20 min at 37 °C. After washing twice with PBS and filtering with a 0.4-μM cell filter, flow cytometry was used to assess intracellular lipid ROS. The iTreg cells were generated in vitro. The transfected HCC cells received coculture with CD4^+^ T cells for 48 h, and proliferation of CD4^+^ T cells received assessment by CFSE staining using flow cytometry. Antibodies including APC-anti-CD4 or PE-Cy7-anti-CD4 APC-anti-CD25, PE-anti-FOXP3, PE-anti-CTLA4, PE-anti-TIGIT, PE-anti-TNFRSF4, or isotype controls provided by Invitrogen (USA) were utilized for analyzing the phenotype of various Tregs. FOXP3/CD25/CTLA4/TIGIT/TNFRSF4 staining was performed with Fix/Perm buffer (Invitrogen, USA). After staining, cells received washing and fixation with 2% paraformaldehyde before analysis through flow cytometry. The results were analyzed using FlowJo software. There are three biological replicates for flow cytometry analysis.

### Iron assay

An iron assay kit was used to detect intracellular Fe^2+^ level or the total iron content (Yu et al. 2023). HCC cells grown in 10-cm^2^ plates (5 × 10^6^ cells/plate) or xenograft tumor tissue sections received treatment with erastin or DMSO for 12 h. After cells or slides received washing with cold PBS, they received mixing with iron assay buffer on ice and centrifugation at 4 °C for 10 min; the supernatant received collection and addition to iron reducer for 30 min. Moreover, the iron probe (100 μL) received addition and mixing, and sample received incubation for 1 h. Finally, a microplate reader was used to assess absorbance at 593 nm. There are three biological replicates for iron assay.

### Xenograft mouse models

Ten SCID or NOD/SCID male mice (4–6 weeks, weight 20 ± 2 g) were obtained from Vital River Laboratory Animal Technology (Beijing, China) and divided into two groups (*n* = 5 per group). The mice were maintained at 24 ± 2 °C under 12-h lighting cycles with free access to food and water. The animal experiments were conducted between June 2022 and July 2022 under the approval of the General Hospital of Ningxia Medical University (ethic approval number: KYLL-2023-0500). Furthermore, 200 μL of HepG2 cells (5 × 10^6^ cells) harboring sh-NC or sh-LINC00942 was subcutaneously injected into right flank of mice. Tumor volume received monitoring every 3 days starting from when it appeared. The mice received sacrificing on the 30th day by cervical dislocation, and tumors were excised and weighed (Wang et al. 2023a). The tumors were stored on ice for subsequent assays. There are five biological replicates for in vivo assays.

### Statistical analysis

Statistical analyses were performed using GraphPad Prism Software (version 8, La Jolla, CA, USA). Student’s *t* test was used to analyze differences between two groups, and one-way ANOVA was used for comparisons of more than two groups. There are three biological replicates for in vitro assays and five biological replicates for in vivo assays. Statistical significance was exhibited when *p* value < 0.05.

## Results

### LINC00942 inhibits ferroptosis and promotes the proliferation of HCC cells

**Fig. 1:**
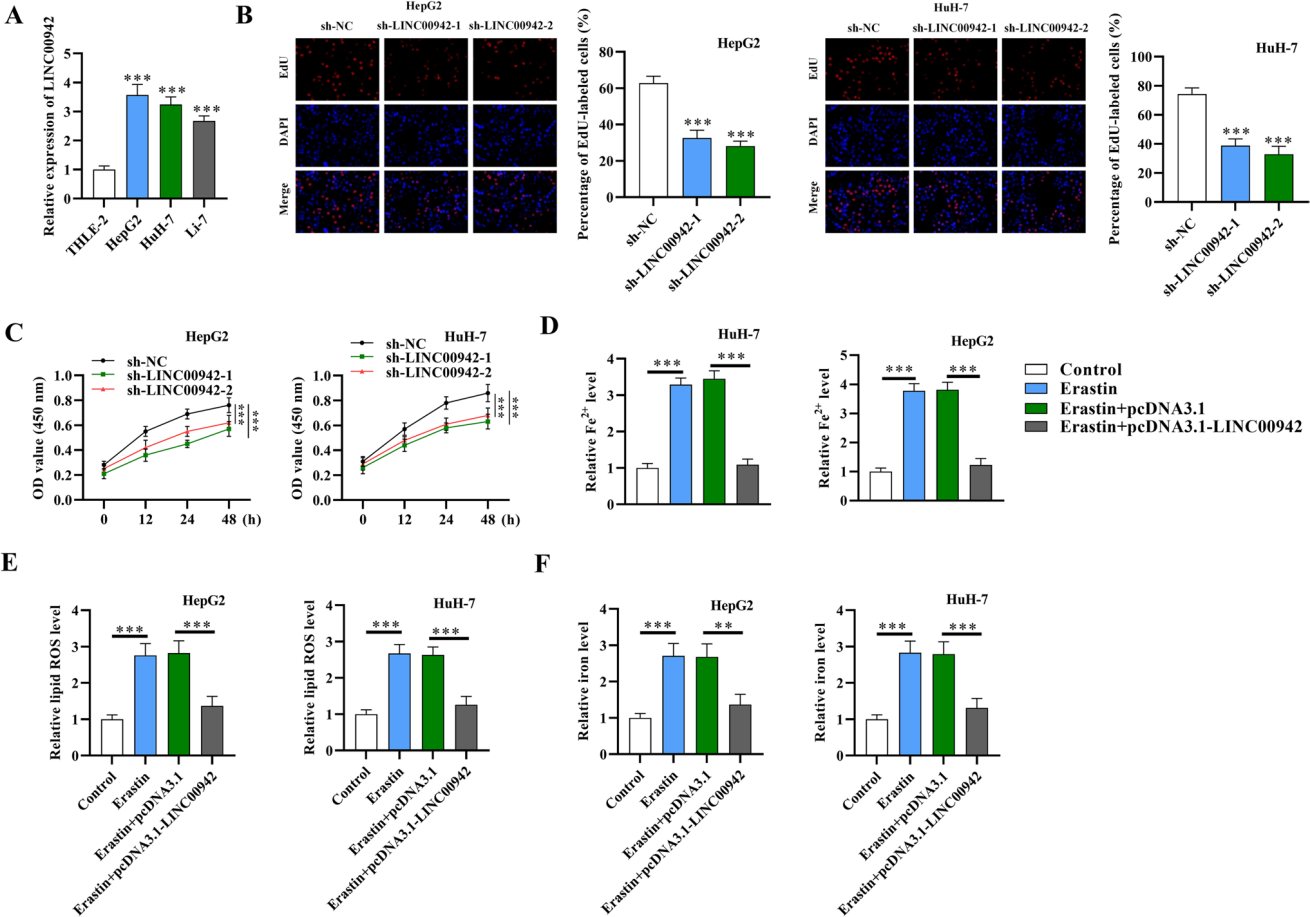
LINC00942 regulates the proliferation and ferroptosis of HCC cells. **A** Comparison of the expression of LINC00942 in HCC cell lines (HepG2, HuH-7, Li-7) and normal adult liver epithelial THLE-2 cells. **B** An EdU assay was conducted to assess HCC cell proliferation after LINC00942 silencing. Scale bar: 50 μm. **C** HCC cell viability was determined using a CCK-8 assay. **D** Relative levels of Fe2^+^ in HCC cells after erastin treatment and LINC00942 overexpression. **E** Flow cytometry analysis was used to detect the intracellular lipid ROS levels in HCC cells after the indicated treatments. **F** The iron levels in HCC cells were quantified using an iron assay kit. **p < 0.01, ***p < 0.001. There are three biological replicates

LINC00942 has been reported as a tumor promoter in various cancers, including lung adenocarcinoma (Xi and Wang 2021), breast cancer (Sun et al. 2020), and gastric cancer (Zhu et al. 2022). However, its explicit function and regulatory mechanism in HCC are unknown. We hypothesized that LINC00942 might promote HCC progression by serving as an oncogene. The effects of LINC00942 on HCC proliferation and ferroptosis were first explored in this study. LINC00942 was demonstrated to be upregulated in HCC cell lines, especially in HepG2 and HuH-7 cells, compared with normal adult liver epithelial THLE-2 cells (Fig. 1A; *p* < 0.001, *n* = 3). The silencing efficiency of sh-LINC00942-1 and sh-LINC00942-2 was validated using qRT-PCR analysis (Supplementary Fig. 1b). As revealed by the EdU and CCK-8 assays, the proliferation and viability of HCC cells were significantly inhibited after LINC00942 knockdown (Fig. 1B, C; *p* < 0.001, *n* = 3). The overexpression efficiency of LINC00942 was verified, as shown in Supplementary Fig. 1C. Because of the critical involvement of Fe^2+^ and lipid ROS in the ferroptosis process (Wang et al. 2017a), we further investigated the accumulation of Fe^2+^, lipid ROS, and iron levels in HCC cells under different treatments. Compared with the control group, the group treated with erastin, a ferroptosis activator, showed a significant increase in the levels of Fe^2+^ in HCC cells, which was reversed by the overexpression of LINC00942 (Fig. 1D; *p* < 0.001, *n* = 3). According to flow cytometry analysis, the intracellular lipid ROS level showed a significant increase in the erastin-treated groups (*p* = 0.0002 in HepG2 cells, *p* = 0.0009 in HuH-7 cells), while LINC00942 overexpression attenuated the erastin-induced increase in lipid ROS levels (Fig. 1E; *p* < 0.001 in HepG2 cells, *p* = 0.0002 in HuH-7 cells, *n* = 3). Similarly, the increase in iron levels by erastin treatment (*p* = 0.0004 in HepG2 cells, *p* = 0.0002 in HuH-7 cells) was attenuated after LINC00942 upregulation (Fig. 1F; *p* = 0.0025 in HepG2 cells, *p* = 0.0008 in HuH-7 cells, *n* = 3). Overall, the results indicate that LINC00942 facilitates HCC cell proliferation and inhibits ferroptosis.

### LINC00942 inhibits HCC ferroptosis by regulating SLC7A11

**Fig. 2:**
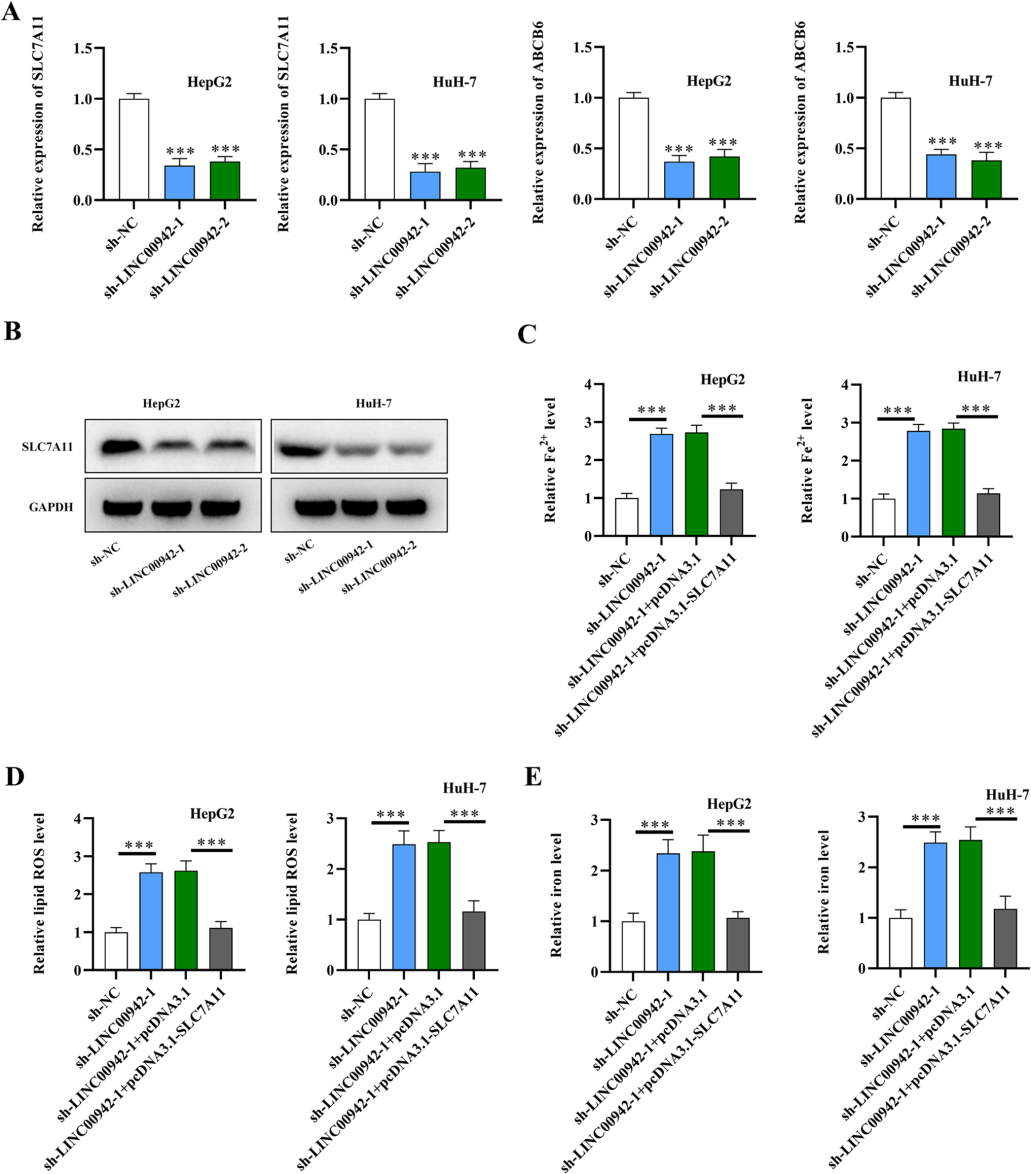
LINC00942 inhibits HCC ferroptosis by regulating SLC7A11. **A** qRT-PCR analysis for quantifying mRNA levels of SLC7A11 and ABCB6 in HCC cells after LINC00942 silencing. **B** Western blot analysis was used to assess protein levels of SLC7A11 in LINC00942-silenced HCC cells. **C–E** Relative Fe^2+^ levels, intracellular lipid ROS levels, and iron levels in HCC cells with LINC00942 deficiency and SLC7A11 overexpression. ***p < 0.001. There are three biological replicates

Based on previous studies (Xu et al. 2021a, b), LINC00942 is proposed to be related to ferroptosis and the immune response in HCC. We further explored the molecular mechanism by which LINC00942 inhibits HCC ferroptosis. Four ferroptosis-related genes (ABCB6, FLVCR1, SLC48A1, and SLC7A11) were previously reported to be involved in the response to immunotherapy in HCC (Tang et al. 2020). Thus, we further investigated the correlation of LINC00942 and the four candidate genes in the GEPIA database. ABCB6 and SLC7A11 were chosen under the condition of *R* > 0.3 (*p* < 0.05) (Supplementary Fig. 2a). Then, we investigated whether LINC00942 regulated the two genes in HCC cells. qRT-PCR analysis showed that both SLC7A11 and ABCB6 were downregulated after LINC00942 silencing, and SLC7A11 exhibited a more significant reduction than ABCB6 (Fig. 2A; *p* < 0.001, *n* = 3). Thus, SLC7A11 was selected for further study. According to the western blot analysis, the protein levels of SLC7A11 were also significantly reduced after LINC00942 knockdown in HCC cells (Fig. 2B). SLC7A11 overexpression efficiency was also validated in HCC cells (Supplementary Fig. 2b). Then, we explored the mechanism by which LINC00942 regulates HCC ferroptosis. The results showed that the increase in relative Fe2^+^ levels, intracellular lipid ROS levels, and iron content induced by LINC00942 deficiency in HCC cells were all reversed by SLC7A11 overexpression (Fig. 2C–E; *p* < 0.001, *n* = 3). These findings suggest that LINC00942 suppresses HCC ferroptosis by upregulating SLC7A11.

### LINC00942 converts naive CD4^+^ T cells to iTreg cells by regulating SLC7A11

**Fig. 3:**
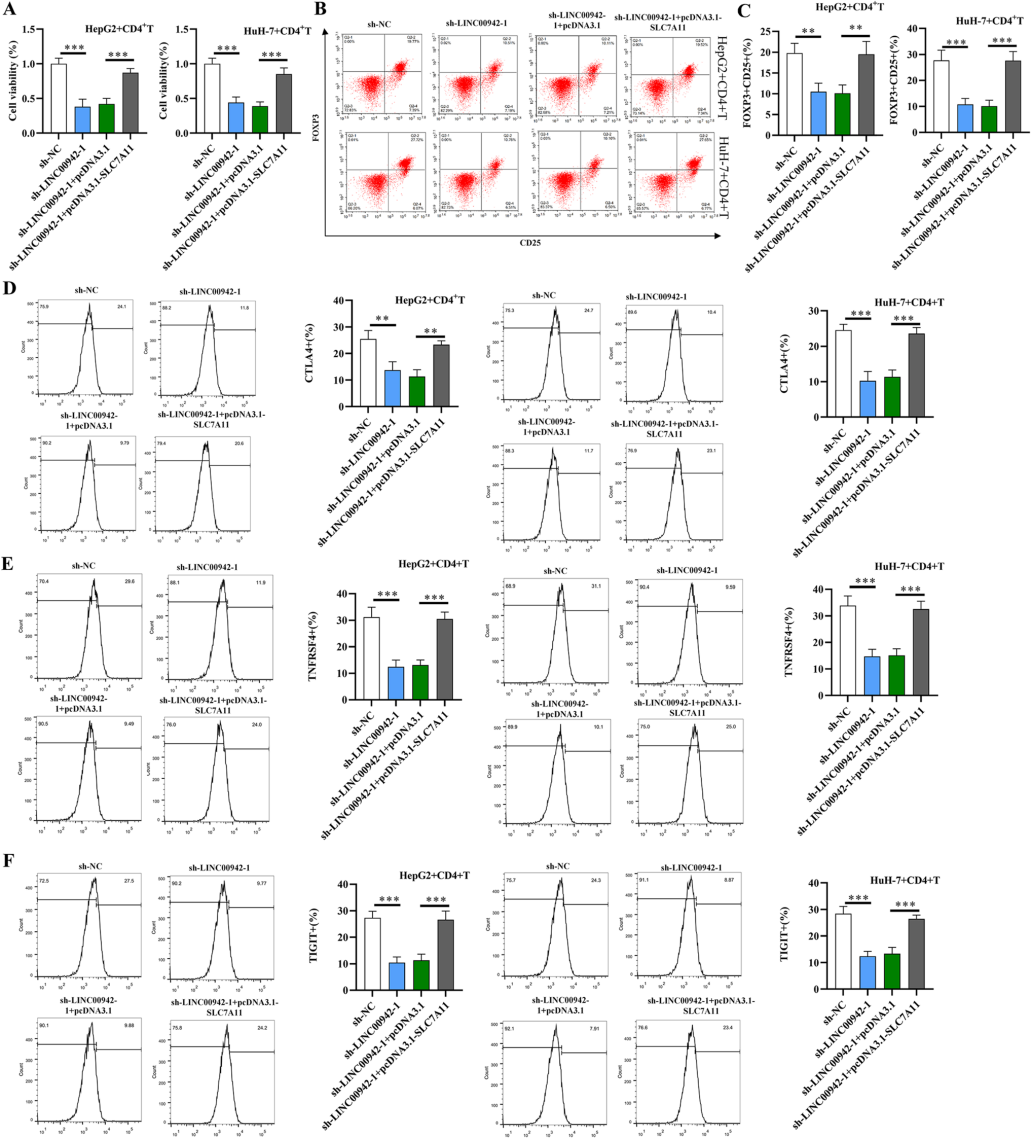
LINC00942 converts naive CD4^+^ T cells to iTreg cells by regulating SLC7A11. **A** Flow cytometry analysis was performed to assess proliferation of CD4^+^ T cells after coincubation with transfected HCC cells for 48 h. **B–F** The effect of LINC0094 knockdown and SLC7A11 overexpression on the expression of Treg cell signature genes (FOXP3/CD25, CTLA4, TNFRSF4, TIGIT) in CD4^+^ T cells after coincubation with transfected HCC cells. **p < 0.01, ***p < 0.001. There are three biological replicates

Previous studies have revealed that LINC00942 is related to the HCC immune response (Xu et al. 2021a, b). Regulatory T cells (Tregs) suppress the immune response against self-structures to prevent autoimmune diseases and are reported to have inhibitory effects on antitumor immunity (Whiteside 2012). FOXP3^+^ T cells are a highly immunosuppressive subset of CD4^+^ T cells and serve as important regulators of the phenotypes and immunosuppressive functions of Treg cells (Togashi et al. 2019). As a functional component of system Xc-, which imports extracellular cystine with intracellular glutamate release at a ratio of 1:1, solute carrier family 7 member 11 (SLC7A11, also known as xCT) acts as an oncogene against oxidative stress and ferroptosis and affects cancer phenotypes and the immune system (Lin et al. 2020b). Recently, SLC7A11 has been reported as a key determinant controlling the proliferation of Treg cells (Procaccini et al. 2021). SLC7A11 has been revealed to defend against oxidative stress and promote cancer proliferation, and its inhibition induces tumor cell death under elevated intracellular ROS. Moreover, SLC7A11 silencing is indicated to elevate lipid ROS and inhibit GSH synthesis in ferroptosis (Koppula et al. 2021). A previous study also suggested that the effect of immunotherapy combined with radiotherapy was enhanced with SLC7A11 inhibition (Lang et al. 2019). Thus, we further investigated whether LINC00942 regulated the proliferation and differentiation of Treg cells by modulating SLC7A11. The transfected HCC cells (HepG2 and HuH-7) were coincubated with CD4^+^ T cells for 48 h. According to flow cytometry analysis, cell proliferation potential was inhibited after LINC00942 silencing, which was reversed by SLC7A11 overexpression (Fig. 3A; *p* < 0.001, *n* = 3). Moreover, LINC00942 knockdown induced a significant reduction in the expression levels of FOXP3/CD25, CTLA4, TNFRSF4, and TIGIT in CD4^+^ T cells after coincubation with transfected HCC cells, and this effect was reversed after SLC7A11 overexpression (Fig. 3B–E; *p* < 0.01, *p* < 0.001, *n* = 3). These results indicated that LINC00942 converts naive CD4^+^ T cells to iTreg cells by regulating SLC7A11.

### IGF2BP3 is a shared RBP for LINC00942 and SLC7A11

**Fig. 4:**
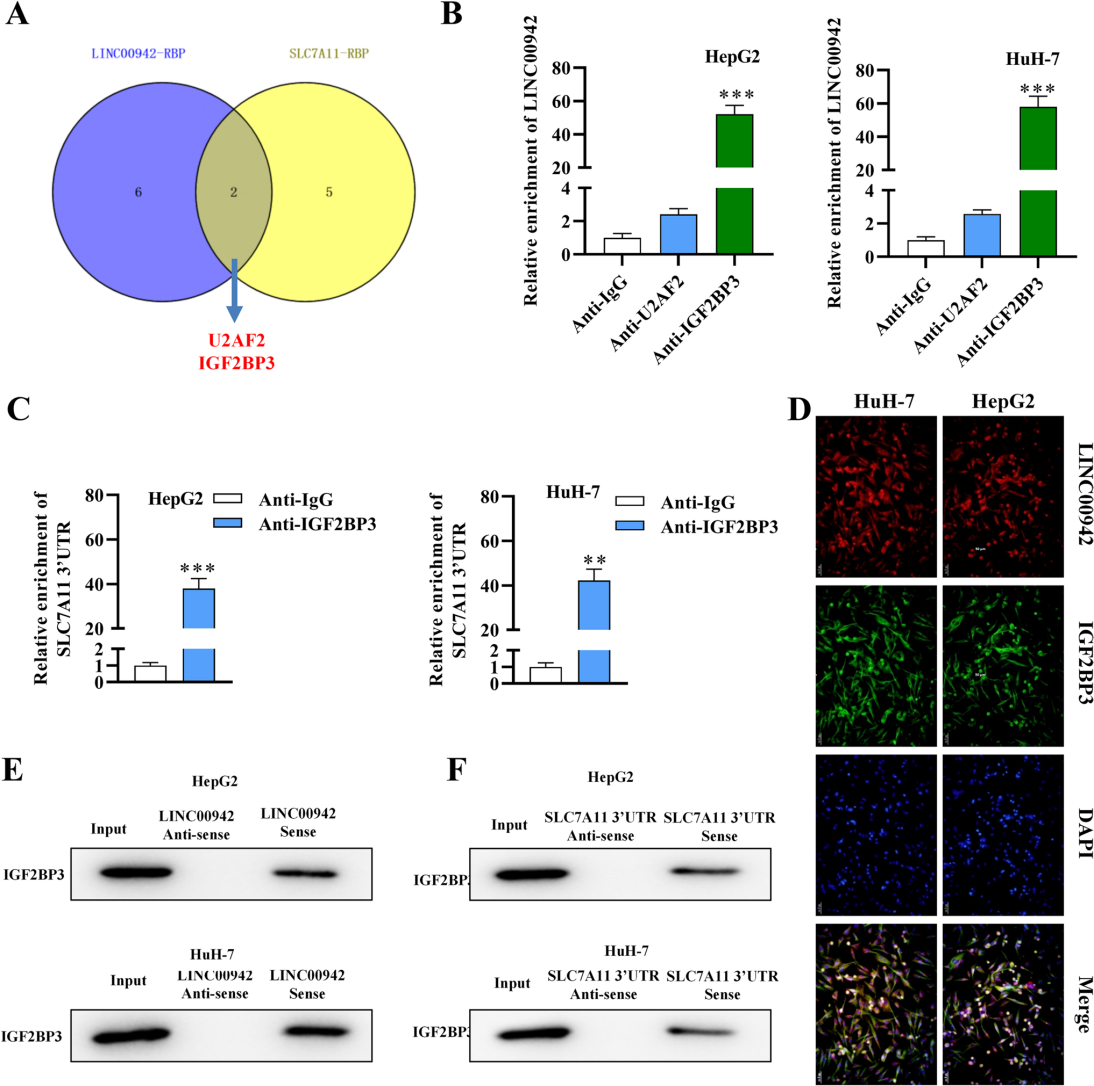
IGF2BP3 is a shared RBP for LINC00942 and SLC7A11. **A** The shared RBPs (U2AF2, IGF2BP3) of LINC00942 on SLC7A11 were revealed by starBase (http://starbase.sysu.edu.cn/). **B** A RIP assay assessed binding of LINC00942 and U2AF2 or IGF2BP3. **C** A RIP assay was used to assess binding of the SLC7A11 3′UTR and IGF2BP3. **D** A FISH assay was conducted to detect colocalization of LINC00942 and IGF2BP3 in HCC cells. Scale bar: 50 μm. **E** and **F** RNA pulldown and western blot assays were used to further explore the binding between IGF2BP3 and LINC00942 or the SLC7A11 3′UTR. **p < 0.01, ***p < 0.001. There are three biological replicates

Next, the regulatory mechanism of LINC00942 on SLC7A11 was explored. The shared RBPs between LINC00942 and SLC7A11 mRNA were searched in starBase. The RBPs for SLC7A11 were selected under CLIP-DaTa ≥ 5, Cluster ≥ 50. The RBPs for LINC00942 were screened under CLIP-DaTa ≥ 1 (Supplementary Fig. 3a). Two shared RBPs (U2AF2 and IGF2BP3) of LINC00942 on SLC7A11 were found (Fig. 4A). According to the RIP assay, LINC00942 was more significantly enriched in the anti-IGF2BP3 precipitates than in the control precipitates; thus, IGF2BP3 was chosen for further study (Fig. 4B; *p* < 0.001, *n* = 3). IGF2BP3 is an RNA N6-methyladenosine (m6A) reader and regulates mRNA stability (Yang et al. 2020). The SRAMP database indicated the m6A sites in the SLC7A11 3′UTR with high confidence (Supplementary Fig. 3b). The RIP assay also revealed that the SLC7A11 3′UTR was highly enriched in the anti-IGF2BP3 precipitates, implying that IGF2BP3 binds with SLC7A11 3′UTR (Fig. 4C; *p* = 0.001 in HepG2 cells, *p* = 0.0044 in HuH-7 cells, *n* = 3). Furthermore, the results of FISH assays demonstrated the colocalization of LINC00942 and IGF2BP3 in HCC cells (Fig. 4D). As revealed by RNA pulldown and western blot assays, IGF2BP3 protein level was evidently attenuated in complexes pulled down by LINC00942 and the SLC7A11 3′UTR (Fig. 4E, F). The results suggested that IGF2BP3 bound to LINC00942 and the SLC7A11 3′UTR in HCC cells.

### LINC00942 recruits IGF2BP3 to promote SLC7A11 mRNA stability in an m6A-dependent manner

**Fig. 5:**
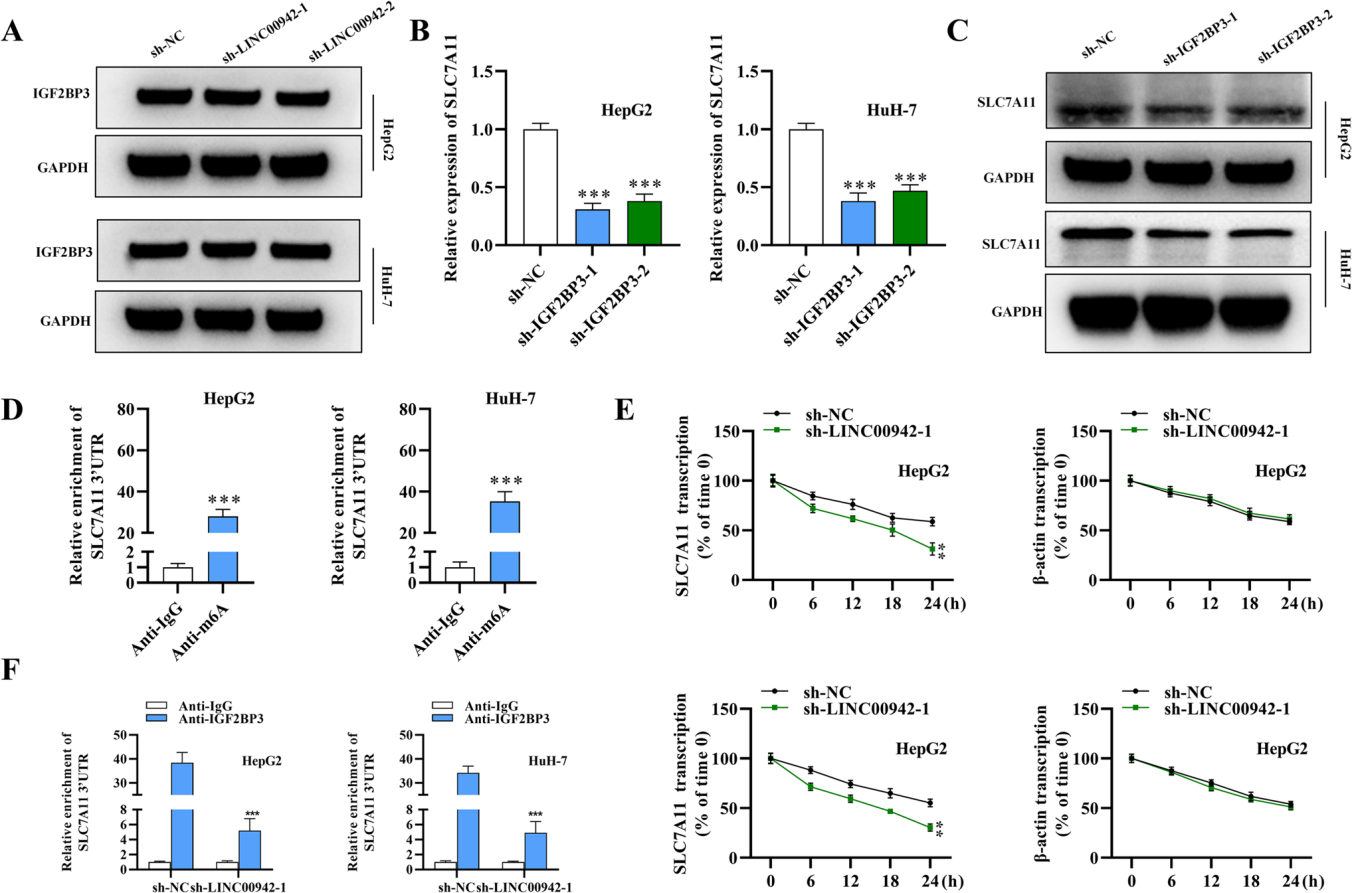
LINC00942 recruits IGF2BP3 to promote SLC7A11 mRNA stability in an m6A-dependent manner. **A** The effect of LINC00942 silencing on IGF2BP3 protein expression was detected by western blotting. **B** and **C** SLC7A11 mRNA expression and protein levels in IGF2BP3-silenced HCC cells. **D** The regulation of SLC7A11 by IGF2BP3 was explored using m6A RNA immunoprecipitation (MeRIP) assays. **E** SLC7A11 mRNA stability was examined in HCC cells treated with 50 mM α-amanitin posttransfection with sh-LINC00942-1. **F** A RIP assay was performed to examine LINC00942 impact on the binding between SLC7A11 and IGF2BP3. **p < 0.01, ***p < 0.001. There are three biological replicates

The explicit mechanism by which LINC00942 regulates SLC7A11 was explored. Insulin-like growth factor 2 mRNA binding protein 3 (IGF2BP3) has been identified as a m6A (N6-methyladenosine) reader that stabilizes methylated mRNAs of oncogenic targets (Mancarella and Scotlandi 2019). IGF2BP3 has been reported to be highly expressed in colon cancer and promote cancer cell proliferation by reading m6A modification of CCND1 (Yang et al. 2020). The m6A modification is at the posttranscriptional level and interprets RNA methylation information and regulates downstream RNA translation and degradation (Wang et al. 2014). The effect of LINC00942 silencing on IGF2BP3 protein expression was assessed using western blotting, and LINC00942 showed no significant effect on IGF2BP3 protein levels in HCC cells (Fig. 5A). Then, we knocked down IGF2BP3 in HCC cells; IGF2BP3 expression was significantly reduced after the transfection of sh-IGF2BP3-1 or sh-IGF2BP3-2 (Supplementary Fig. 3c). As shown in Fig. 5B, C (*p* < 0.001, *n* = 3), the SLC7A11 mRNA expression and protein levels showed a significant decrease after silencing IGF2BP3 in HCC cells. Since we found m6A sites in the SLC7A11 3′UTR with high confidence (Supplementary Fig. 3b), we further investigated whether IGF2BP3 regulated SLC7A11 through m6A modification using MeRIP assays. The results showed that the SLC7A11 3′UTR was enriched in the anti-m6A precipitates, which suggested an interaction between IGF2BP3 and SLC7A11 (Fig. 5D; *p* = 0.0002, *n* = 3). Then, the mRNA stability of SLC7A11 was examined in HCC cells treated with 50 mM actinomycin D. SLC7A11 transcription was inhibited after LINC00942 silencing compared with that in the β-actin group (Fig. 5E; *p* = 0.0030 in HepG2 cells, *p* = 0.0012 in HuH-7 cells, *n* = 3). Furthermore, RIP assays showed that LINC00942 silencing significantly reduced the enrichment of the SLC7A11 3′UTR in the anti-IGF2BP3 precipitates, which indicated that LINC00942 promotes the binding between SLC7A11 and IGF2BP3 (Fig. 5F; *p* < 0.001, *n* = 3). Overall, these results revealed that LINC00942 recruits IGF2BP3 to increase the mRNA stability of SLC7A11 in an m6A-dependent manner.

### LINC00942 inhibits ferroptosis and induces immunosuppression via Tregs

**Fig. 6:**
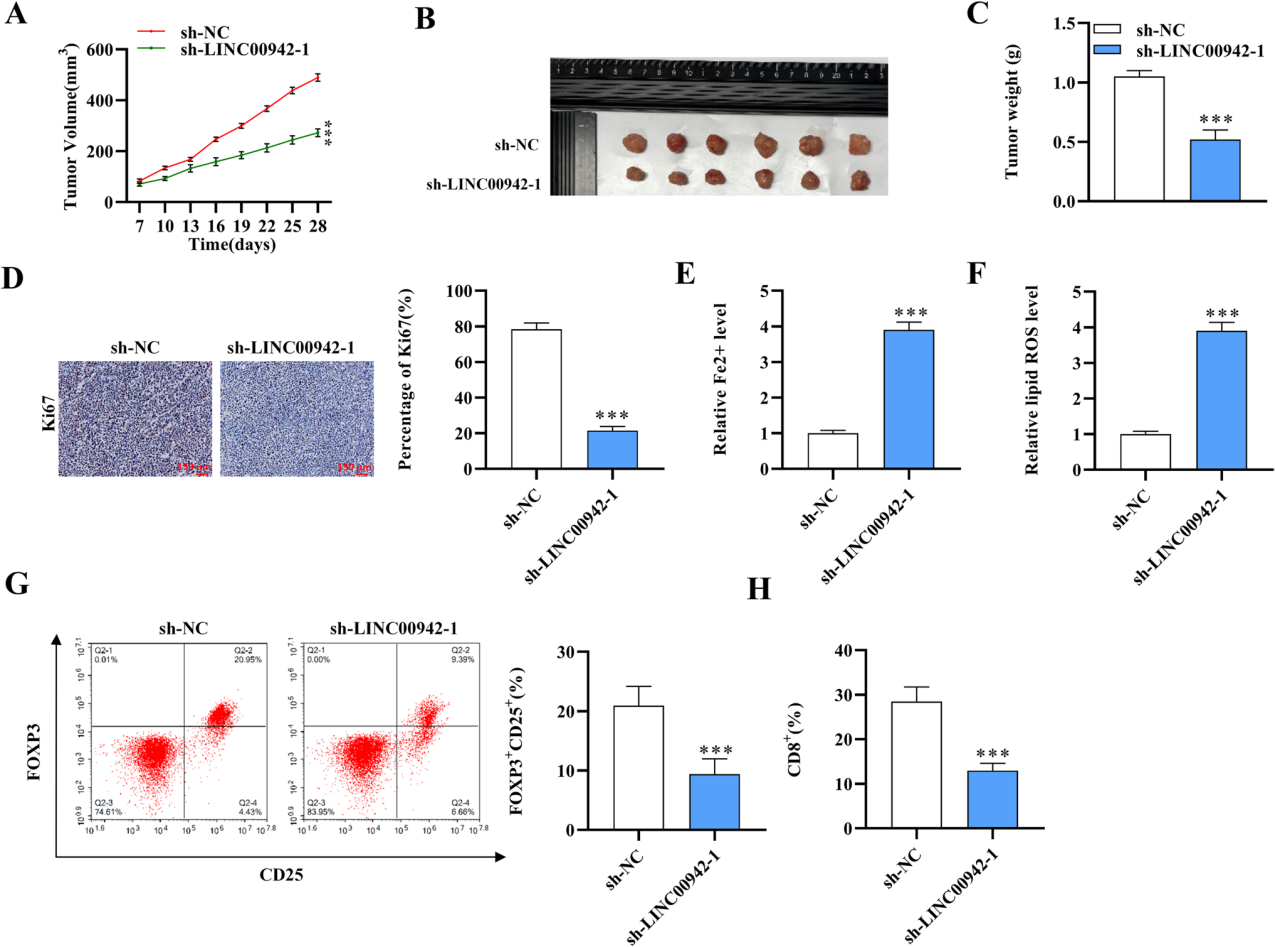
LINC00942 inhibits ferroptosis and induces immunosuppression via Tregs. **A** The tumor volume was assessed every 3 days beginning on day 7 in the sh-NC and sh-LINC00942-1 groups. *N* = 5. **B** Images of mouse tumors in the sh-NC or sh-LINC00942-1 groups. *N* = 5. **C** The mouse tumor weight in the two groups. *N* = 5. **D** Ki-67 expression in tumor tissues was assessed using immunohistochemistry. *N* = 5. Scale bar: 150 μm. **E** and **D** Fe^2+^ levels and intracellular lipid ROS levels in mouse tumor tissues. *N* = 5. **G** and **H** FOXP3^+^CD25^+^ and CD8^+^ Treg cells in mouse tumors. *N* = 5. ***p < 0.001. There are five biological replicates

Next, we explored the function and mechanism of LINC00942 in HCC in vivo. The xenograft mouse models were successfully established by inoculating HepG2 cells transfected with sh-NC and sh-LINC00942-1. The tumor volume and growth rate were significantly decreased after the knockdown of LINC00942 (Fig. 6A; *p* < 0.001, *n* = 5). The tumor size and weight were also evidently declined in the sh-LINC00942-1 groups (Fig. 6B, C; *p* = 0.0006, *n* = 5). Ki-67 expression was reduced after LINC00942 silencing in HCC tumor tissues (Fig. 6D; *p* < 0.001, *n* = 5). Moreover, the Fe^2+^ levels and intracellular lipid ROS levels in mouse tumor tissues showed a marked elevation, while the numbers of FOXP3^+^CD25^+^ and CD8^+^ Treg cells in tumors were reduced, which revealed that LINC00942 inhibits ferroptosis and induces immunosuppression (Fig. 6E–H; *p* < 0.001, *n* = 5).

**Fig. 7:**
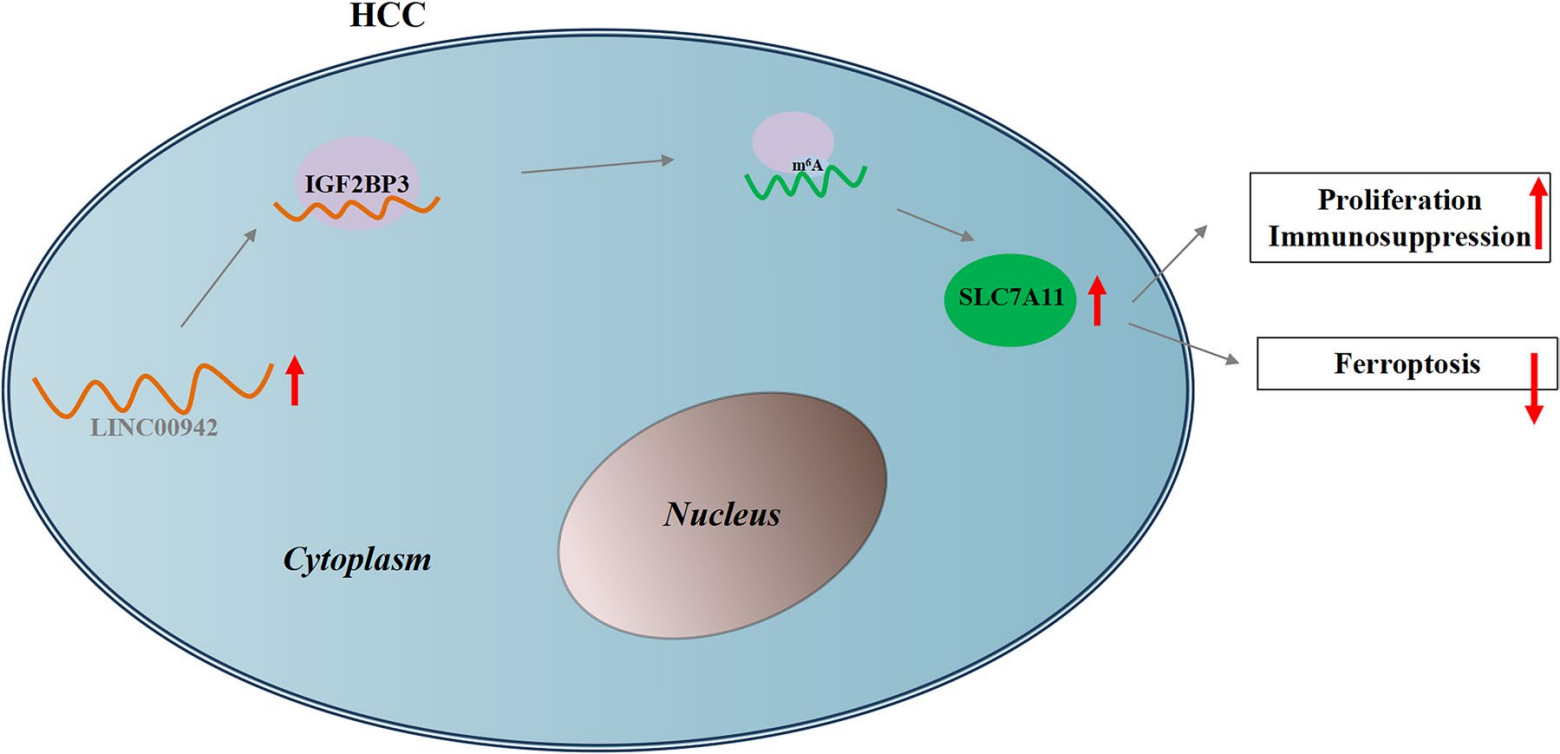
Graphic abstract

Collectively, LINC00942 was demonstrated to promote HCC cell proliferation, inhibit ferroptosis, and trigger immunosuppression through regulating SLC7A11 in a m6A-dependent manner mediated by IGF2BP3 (Fig. 7).

## Discussion

Previously, silencing of lncRNA DUXAP8 was found to synergistically enhance sorafenib-induced ferroptosis in HCC through SLC7A11 depalmitoylation (Shi et al. 2023). HDLBP-stabilized lncFAL suppresses ferroptosis vulnerability by diminishing Trim69-dependent FSP1 degradation in HCC (Yuan et al. 2022). LncRNAs can mediate ferroptosis in HCC in an RBP-dependent manner. Nevertheless, there are few reports on the roles of immunosuppression of regulatory T cells in lncRNA-mediated ferroptosis in HCC. In this study, we clarified the function and mechanism of LINC00942 in the progression of HCC in vitro and in vivo. LINC00942 was demonstrated to promote HCC cell proliferation, inhibit ferroptosis, and convert naive CD4^+^ T cells to iTreg cells by regulating SLC7A11. The mechanism by which LINC00942 regulates SLC7A11 was further explored. We found that IGF2BP3 was a RBP for LINC00942 and SLC7A11 and that LINC00942 recruited IGF2BP3 to promote SLC7A11 mRNA stability in an m6A-dependent manner. Xenograft mouse models were established, and experiment results revealed that LINC00942 suppressed HCC ferroptosis and induced immunosuppression via Tregs in vivo. Thus, our research supplemented findings on roles of the immunosuppression of regulatory T cells in lncRNA-RBP functions in HCC.

LINC00942 has been reported as a tumor promoter in various cancers, including lung adenocarcinoma (Xi and Wang 2021), breast cancer (Sun et al. 2020), and gastric cancer (Zhu et al. 2022). However, its explicit function and regulatory mechanism in HCC are largely unknown. Based on previous studies (Xu et al. 2021a, b), LINC00942 is proposed to be related to ferroptosis and the immune response in HCC. In this study, we found that LINC00942 was upregulated in HCC tissues and cells. LINC00942 deficiency suppressed HCC cell proliferation. The erastin-induced increase in intracellular lipid ROS levels and iron content was significantly reversed after LINC00942 overexpression. Regulatory T cells (Tregs) suppress the immune response against self-structures to prevent autoimmune diseases and are reported to have inhibitory effects on antitumor immunity in certain settings (Whiteside 2012). FOXP3^+^ T cells are a highly immunosuppressive subset of CD4^+^ T cells and serve as important regulators of the phenotypes and immunosuppressive functions of Treg cells (Togashi et al. 2019). We found that LINC00942 inhibited HCC ferroptosis and converted naive CD4^+^ T cells to iTreg cells in vitro. The number of FOXP3^+^CD25^+^CD4^+^ T cells was significantly decreased after LINC00942 silencing in HCC cells. Results form in vivo assays also revealed that LINC00942 suppressed HCC tumor cell proliferation and tumor growth and induced immunosuppression by inducing Treg cells.

SLC7A11 acts as an oncogene against oxidative stress and ferroptosis and affects cancer phenotypes and the immune system (Lin et al. 2020b). It has been revealed to defend against oxidative stress and promote cancer proliferation, and its inhibition induces tumor cell death under elevated intracellular ROS. Moreover, SLC7A11 silencing is indicated to elevate lipid ROS and inhibit GSH synthesis in ferroptosis (Koppula et al. 2021). A previous study also suggested that the effect of immunotherapy combined with radiotherapy was enhanced with SLC7A11 inhibition (Lang et al. 2019). In our study, we also found that LINC00942 suppressed ferroptosis by regulating SLC7A11. LINC00942 expression was revealed to be positively correlated with SLC7A11 in HCC tissues. SLC7A11 mRNA and protein levels were also decreased in HCC cells with silenced LINC00942. The inhibition of HCC ferroptosis and iTreg differentiation induced by LINC00942 deficiency were reversed by SLC7A11 overexpression.

IGF2BP3 has been identified as an m6A (N6-methyladenosine) reader that stabilizes methylated mRNAs of oncogenic targets (Mancarella and Scotlandi 2019). IGF2BP3 has been reported to be highly expressed in colon cancer and promote cancer cell proliferation by reading m6A modification of CCND1 (Yang et al. 2020). m6A modification occurs at the posttranscriptional level, and readers interpret RNA methylation information and regulate downstream RNA translation and degradation (Wang et al. 2014). In our study, we found that IGF2BP3 was a RBP for LINC00942 and SLC7A11, and LINC00942 did not affect IGF2BP3 expression in HCC cells. However, the mRNA and protein levels of SLC7A11 were negatively modulated by IGF2BP3. Moreover, the binding between IGF2BP3 and the SLC7A11 3′UTR was verified, and LINC00942 was indicated to facilitate the interaction between the SLC7A11 3′UTR and IGF2BP3. Thus, LINC00942 was suggested to recruit IGF2BP3 to promote SLC7A11 mRNA stability in an m6A-dependent manner.

However, there are still some limitations to this research. It has been revealed that lncRNAs exert biological functions through the miRNA/mRNA axis. Our research did not explore whether LINC00942 regulates SLC7A11 in HCC in such a pattern. Moreover, we did not perform rescue assays in vivo to further verify the regulatory pattern of LINC00942 underlying ferroptosis and immunosuppression in HCC. In our future research, these topics will need further exploration; moreover, downstream ferroptosis-related signaling pathways mediated by LINC00942 also need further exploration.

## Conclusion

LINC00942 suppresses ferroptosis and induces immunosuppression via Tregs in HCC by recruiting IGF2BP3 to promote SLC7A11 mRNA stability in an m6A-dependent manner. These findings may provide novel therapeutic targets for the treatment of HCC.

### Supplementary information

Below is the link to the electronic supplementary material.

Supplementary file1 (DOCX 661 KB)

## Data Availability

The datasets during and/or analyzed during the current study available from the corresponding author on reasonable request.
