# Supplementary material for: LINC00942 inhibits ferroptosis and induces the immunosuppression of regulatory T cells by recruiting IGF2BP3/SLC7A11 in hepatocellular carcinoma
Source: Funct Integr Genomics. 2024 Feb 14;24(1):29. doi: 10.1007/s10142-024-01292-4 (PMC10867055; doi:10.1007/s10142-024-01292-4)
Supplement: Supplementary file 1 — Supplementary file1 (DOCX 661 KB) [file 10142_2024_1292_MOESM1_ESM.docx]

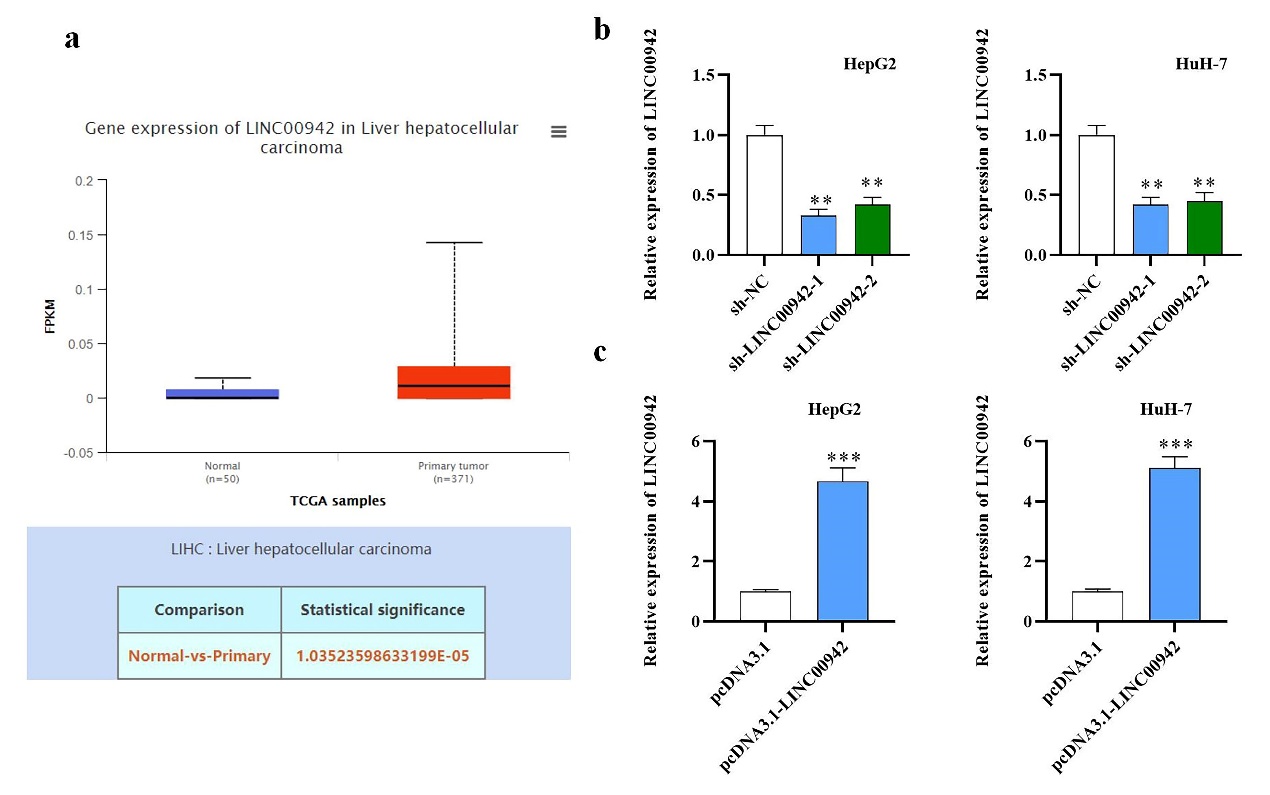


**Supplementary Figure 1**

(A) UALCAN database was used to analyze the expression of LINC00942 in normal human liver tissues and HCC tumor tissues. (B) The expression of LINC00942 posttransfection of sh-LINC00942-1 and sh-LINC00942-2 in HCC cells. (C) The overexpression efficiency of LINC00942 was detected using qRT-PCR analysis. **p<0.01, ***p<0.001.


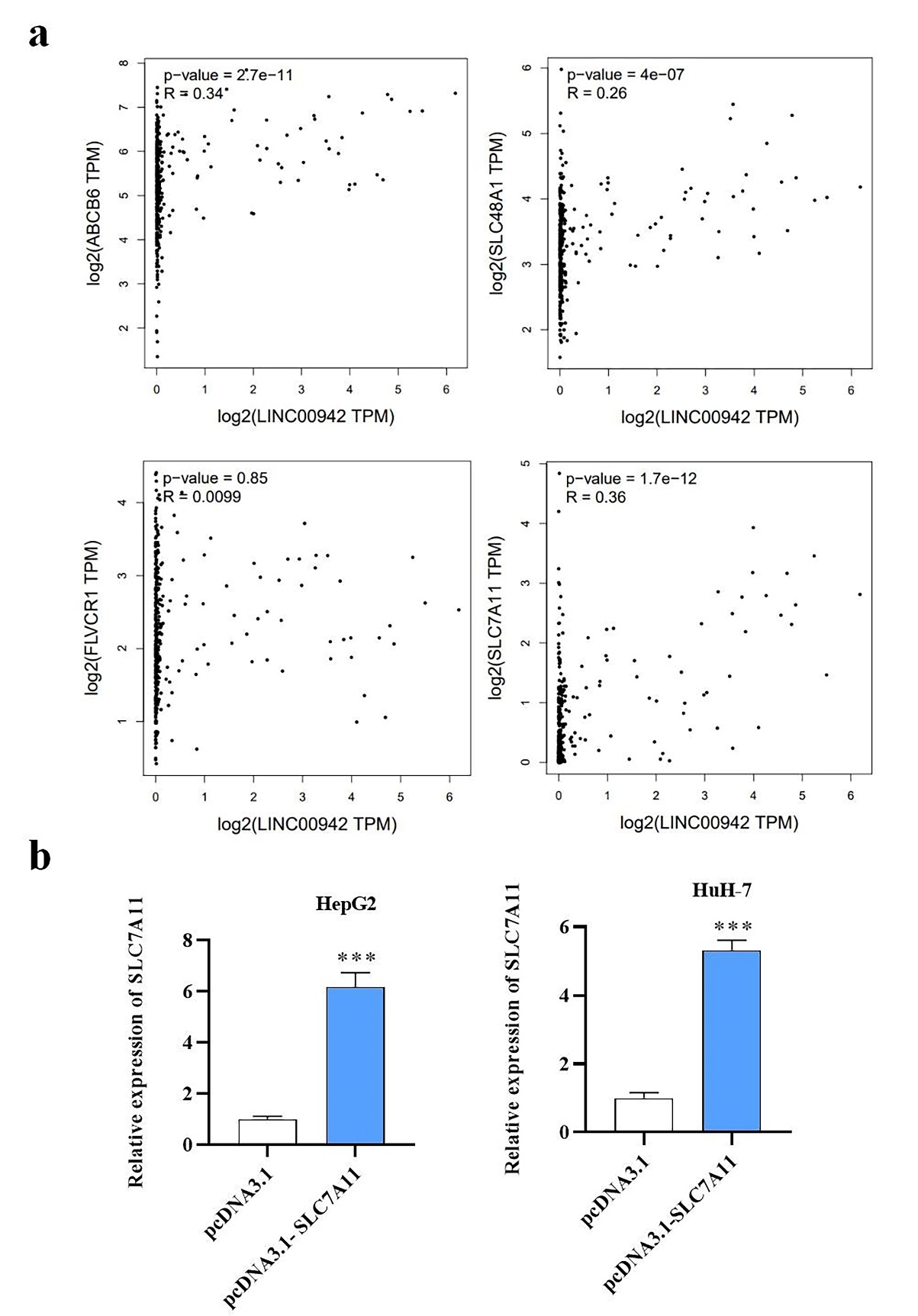


**Supplementary Figure 2**

(A) The correlation of LINC00942 and four candidate genes (ABCB6, SLC48A1, FLVCR1, SLC7A11) in the GEPIA database. (B) SLC7A11 overexpression efficiency in HCC cells was determined by qRT-PCR. ***p<0.001.


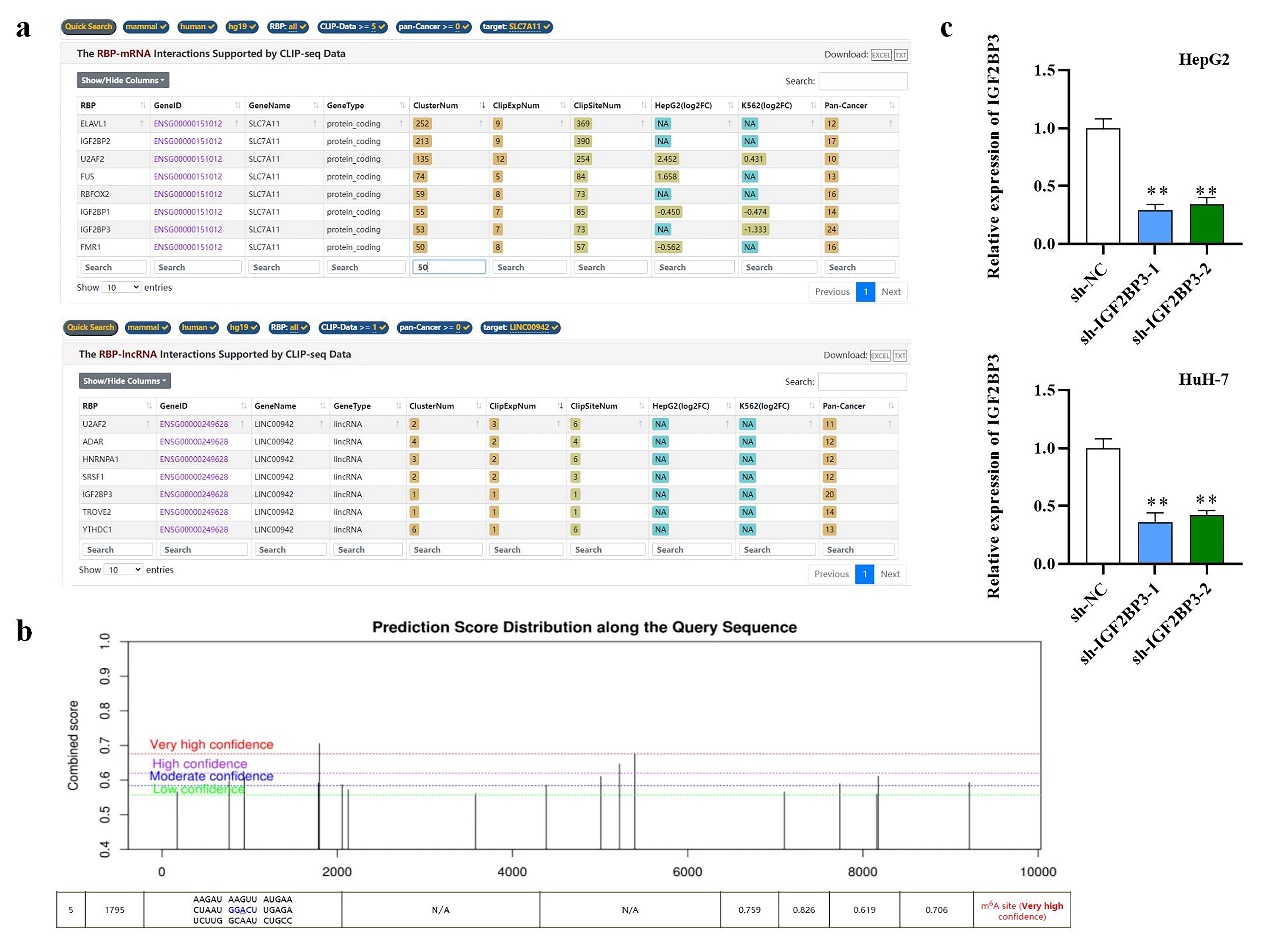


**Supplementary Figure 3**

(A) StarBase showed the RBP for SLC7A11 mRNA under CLIP-DaTa≥5, Cluster≥50; RBP for LINC00942 CLIP-DaTa≥1. (B) SRAMP database predicted the m6A site of SLC7A11. (C) The knockdown efficiency of IGF2BP3 in HCC cells was assessed using qRT-PCR. **p<0.01.
